# Selection of an Appropriate In Vitro Susceptibility Test for Assessing Anti-*Pythium insidiosum* Activity of Potassium Iodide, Triamcinolone Acetonide, Dimethyl Sulfoxide, and Ethanol

**DOI:** 10.3390/jof8111116

**Published:** 2022-10-24

**Authors:** Hanna Yolanda, Tassanee Lohnoo, Thidarat Rujirawat, Wanta Yingyong, Yothin Kumsang, Pattarana Sae-Chew, Penpan Payattikul, Theerapong Krajaejun

**Affiliations:** 1 Program in Translational Medicine, Faculty of Medicine, Ramathibodi Hospital, Mahidol University, Bangkok 10400, Thailand; 2 Department of Parasitology, School of Medicine and Health Sciences, Atma Jaya Catholic University of Indonesia, Jakarta 14440, Indonesia; 3 Research Center, Faculty of Medicine, Ramathibodi Hospital, Mahidol University, Bangkok 10400, Thailand; 4 Department of Pathology, Faculty of Medicine, Ramathibodi Hospital, Mahidol University, Bangkok 10400, Thailand

**Keywords:** pythiosis, *Pythium insidiosum*, oomycete, in vitro drug susceptibility, treatment

## Abstract

The orphan but highly virulent pathogen *Pythium insidiosum* causes pythiosis in humans and animals. Surgery is a primary treatment aiming to cure but trading off losing affected organs. Antimicrobial drugs show limited efficacy in treating pythiosis. Alternative drugs effective against the pathogen are needed. In-house drug susceptibility tests (i.e., broth dilution, disc diffusion, and radial growth assays) have been established, some of which adapted the standard protocols (i.e., CLSI M38-A2 and CLSI M51) designed for fungi. Hyphal plug, hyphal suspension, and zoospore are inocula commonly used in the drug susceptibility assessment for *P. insidiosum*. A side-by-side comparison demonstrated that each method had advantages and limitations. Minimum inhibitory and cidal concentrations of a drug varied depending on the selected method. Material availability, user experience, and organism and drug quantities determined which susceptibility assay should be used. We employed the hyphal plug and a combination of broth dilution and radial growth methods to screen and validate the anti-*P. insidiosum* activities of several previously reported chemicals, including potassium iodide, triamcinolone acetonide, dimethyl sulfoxide, and ethanol, in which data on their anti-*P. insidiosum* efficacy are limited. We tested each chemical against 29 genetically diverse isolates of *P. insidiosum*. These chemicals possessed direct antimicrobial effects on the growth of the pathogen in a dose- and time-dependent manner, suggesting their potential application in pythiosis treatment. Future attempts should focus on standardizing these drug susceptibility methods, such as determining susceptibility/resistant breakpoints, so healthcare workers can confidently interpret a result and select an effective drug against *P. insidiosum*.

## 1. Introduction

The filamentous organism *Pythium insidiosum* is a member of the oomycetes that belong to the Kingdom Stramenopiles and causes a fatal infectious condition called pythiosis in humans and animals [1]. The number of pythiosis cases has been increasingly documented in tropical and subtropical countries [2]. *P. insidiosum* inhabits freshwater, where it colonizes a water plant and produces zoospores to complete its life cycle [3,4,5,6]. Clinical manifestations of pythiosis include cutaneous granulomatous ulcers [7,8], gastrointestinal lesion [9,10], corneal ulcers [11,12], arteritis [13,14], and disseminated infection [15,16]. A definitive diagnosis of pythiosis relies on culture-based identification [17,18,19,20], histopathological examination [21,22], serological assays [23,24,25,26,27], molecular methods [28,29,30,31,32,33], and proteomic assessment [34,35].

The overall mortality rate in humans and animals with pythiosis is ~30% [2]. Antimicrobial medication (i.e., itraconazole, terbinafine, amphotericin B, linezolid, azithromycin, and doxycycline) is usually ineffective in pythiosis treatment [36,37,38,39,40,41,42,43,44,45,46,47,48,49,50]. However, some ocular pythiosis patients showed a favorable response after such medical treatment. Surgical removal of an infected organ is the primary treatment for a pythiosis patient [51,52,53,54,55]. Administration of the immunotherapeutic antigen extracted from *P. insidiosum* could reduce disease morbidity and mortality [56,57,58,59,60,61,62]. In some pythiosis patients, surgical intervention is impossible or fails to remove all infected tissue [63,64,65,66]. The management of such cases relies on antimicrobial agents and other treatment modalities to control the disease and prevent a recurrence. Some unconventional medications (i.e., potassium iodide (KI) [15,39,46,67,68,69,70,71,72,73,74,75,76,77,78], triamcinolone acetonide (TA) [68,79,80,81,82], dimethyl sulfoxide (DMSO) [83,84,85,86,87], and ethanol (EtOH) [88,89,90]) have been implemented for the management of several pythiosis patients and show satisfactory treatment outcomes.

KI, TA, DMSO, and EtOH were not originally designed for use as antimicrobial agents. Their information regarding the anti-*P. insidiosum* effect is limited and has not been comprehensively explored. KI is an inorganic salt used for many purposes. In the industry, it has been applied for inhibiting corrosion, facilitating chemical transformation, and catalyzing biodiesel [91,92,93]. In medicine, this chemical has been utilized in various clinical conditions, such as protecting the thyroid gland against the iodine-131 radioisotope, controlling inflammation in dermatoses, and treating several mycoses (i.e., sporotrichosis, cryptococcosis, entomophthoramycosis, and pythiosis) [94,95,96]. Administration of KI, as a part of the treatment, led to a clinical improvement in some humans, horses, and sheep with cutaneous pythiosis [15,39,46,67,69,72,75,77,78,97]. Additionally, KI has been used in treating other forms of pythiosis (i.e., vascular, ocular, disseminated) [54,69,70,71,74,77,98,99,100].

Steroids (i.e., TA, prednisone, and dexamethasone) modulate the immune response, an important property used for controlling many medical conditions, such as allergies, autoimmune diseases, and inflammatory disorders [101,102,103]. Regarding pythiosis treatment, TA has been used as a monotherapy in some affected horses that fully recovered from the disease [68,79,80,81]. When combined with surgery, immunotherapy, and other medications (i.e., terbinafine, itraconazole, and mefenoxam), prednisone successfully cured dogs with pythiosis [63,104]. However, prednisone and dexamethasone in treating human pythiosis led to disease progression [38,53,66,76]. The direct and indirect effects of steroids against *P. insidiosum* need further exploration to understand its underlying antimicrobial mechanism.

DMSO is a solvent used for preparing chemical solutions [105]. It possesses anti-inflammatory, antioxidant, and antimicrobial properties [106,107,108,109]. DMSO was combined with amphotericin B in treating horses with pythiosis [87]. It was also post-surgically administered as an adjunctive treatment in several affected horses [86]. Such clinical applications of DMSO were associated with improved treatment outcomes in all 32 affected horses [86,87]. DMSO is commonly used to solubilize a test drug for in vitro susceptibility studies of *P. insidiosum* [110,111,112,113]. A direct effect of DMSO against *P. insidiosum* should be assessed for its possible interference in the interpretation of drug susceptibility results and its potential clinical use in pythiosis treatment.

EtOH is an antiseptic agent with a broad antimicrobial activity [114,115,116]. It has been used as a part of the treatment during ocular surgery, such as laser in situ keratomileusis (LASIK) [117], management of iris cyst [118,119], and periorbital arteriovenous malformation [120]. In addition to surgery, medication, and cryotherapy, absolute EtOH was topically applied to treat and prevent recurrent infection in some patients with *Pythium* keratitis [88,89,90]. An initial assessment showed that as low as 20% EtOH could markedly inhibit the in vitro growth of a clinical isolate of *P. insidiosum* [88]. Due to its reported safety and anti-*P. insidiosum* activity, using EtOH as an adjunctive treatment to improve the clinical treatment outcome of ocular pythiosis patients, is promising [89,90].

As mentioned above, KI, TA, DMSO, and EtOH could be alternative medications for treating pythiosis. However, information on their antimicrobial effects against biologically diverse isolates of *P. insidiosum* is lacking. Regarding in vitro drug susceptibility evaluation, there is no standardized method for *P. insidiosum*. Several in-house methods have been established, which can be divided into agar-based (i.e., radial growth and disc diffusion) and broth-based (i.e., broth dilution) techniques, employing various inoculum types (i.e., hyphal plug, hyphal suspension, and zoospores) [121,122,123,124,125,126,127,128,129]. Other than procedure duration and complexity, a different susceptibility method or inoculum type could provide a different result, particularly minimal inhibitory concentration (MIC) [111,130]. The current study aims to (i) compare various in vitro susceptibility assays for their advantages and disadvantages and (ii) comprehensively assess the anti-*P. insidiosum* activity of KI, TA, DMSO, and EtOH using an appropriate in vitro susceptibility assay. This study suggests how drug susceptibility assessment for *P. insidiosum* can be selected and performed in a clinical laboratory and describes the potential use of KI, TA, DMSO, and EtOH in pythiosis treatment.

## 2. Materials and Methods

### 2.1. Microorganisms

Twenty-nine *P. insidiosum* isolates were tested for their drug susceptibility against KI, TA, DMSO, and EtOH. Associated information regarding the affected host, infected tissue, country of origin, and phylogenetic group (clade) of the pathogens are provided in Table 1. The identity of each isolate was confirmed using PCR and sequence homology analysis [28,29,30,33,131,132,133,134,135,136]. All organisms were maintained on Sabouraud dextrose (SD) agar (1% peptone (Gibco Thermofisher, Detroit, MI, USA), 2% glucose (Himedia, Maharashtra, India), 1.2% agar (Difco BD, Le Pont de Claix, France), and distilled water) and subcultured monthly until use. Each agar plate was prepared by pouring 20 mL of the sterile warm SD medium (pH 7.2) into a 9 cm diameter petri dish and letting it set at room temperature.

### 2.2. Preparation of an Inoculum

Three inoculum types (i.e., hyphal plug, hyphal suspension, and zoospore) were prepared for in vitro drug susceptibility analysis of *P. insidiosum*. Hyphal plugs were excised using a Cork borer (5 mm in diameter) from the edge of a *P. insidiosum* colony (7 days of age) actively growing on SD agar and used as an inoculum as described elsewhere [122,137]. For the agar-based susceptibility assays (i.e., radial growth and disc diffusion), the organism side of each hyphal plug was faced down onto an SD agar plate containing a drug of choice [121,137]. The hyphal suspension was prepared by scraping the surface of a *P. insidiosum* colony on an SD agar plate in the presence of 10 mL of sterile distilled water. The resulting hyphal suspension was adjusted to 80–85% transmittance using a spectrophotometer (at 530-nm wavelength) and diluted to 1:10 in SD broth [1% peptone (Gibco Thermofisher, Detroit, MI, USA), 2% glucose (Himedia, Maharashtra, India), and distilled water; pH 7.2] [121,128,130].

Zoospores, the asexual stage of *P. insidiosum*, were generated following the previously described methods with some modifications [121,138,139]. Briefly, a hyphal plug was placed on SD agar and co-incubated with sterile grass leaves at 37 °C for 24 h. The grass leaves were transferred to a 50-mL beaker and submerged in 20 mL of the induction medium, which is a mixture of 0.5 mL solution A (1 M K_2_HPO_4_, 1 M KH_2_PO_4_, and 1 M (NH_4_)_2_HPO_4_), 0.1 mL solution B (0.5 M MgCl_2_·6H_2_O and 0.5 M CaCl_2_·2H_2_O), and 1000 mL sterile distilled water [121,138,139]. The released zoospores (usually observed within 8–12 h) were quantitated using a hemocytometer, and cell density was adjusted to 2–3 × 10^3^ cells/mL for broth dilution assay and 3–5 × 10^4^ cells/mL for disc diffusion analysis (see below) [112,121,129,140,141].

### 2.3. In Vitro Susceptibility Assays

Three in vitro susceptibility methods were used in this study: broth dilution, radial growth assay, and disc diffusion. The selection of inoculum types for each assay relied on the previously reported susceptibility assays [111,112,121,123,130]. The broth dilution assay was performed using either multiple tubes (i.e., 5 mL test tubes) or a 24-well plate containing *P. insidiosum* inoculum (i.e., hyphae plug, hyphae suspension or zoospore) in SD broth at various drug concentrations [111,126,130,142]. A susceptibility readout relied on the presence (growth/resistant) or absence (no growth/susceptible) of a growing colony by the naked eye.

The radial growth assay was conducted using a set of SD agar plates containing various drug concentrations. A test drug was added to the desired concentration in warm SD agar (~56 °C), mixed well, poured (20 mL) into a 9 cm diameter petri dish, and let the plate settle at room temperature [111,122,125,137]. A hyphal plug containing an actively growing *P. insidiosum* colony was placed face down on a drug-containing SD agar [122,125], incubated at 37 °C, and checked for radial growth daily for 2 days. The result was reported as a relative percent radial growth of a *P. insidiosum* colony in a drug-containing agar compared with that in a drug-free medium (control) [111,122,125].

Disc diffusion assay employed a 6 mm sterile paper disc (grade AA discs, Whatman^TM^, GE Healthcare Life Sciences, Buckinghamshire, UK) containing 20 μL of a test drug at the desired concentration [123,143,144,145]. The drug-soaked disc was placed 2 cm away from a hyphae plug which was inoculated in a way that the organism directly contacted a plain SD agar [121]. A clear zone (*P. insidiosum* inhibition zone) around the disc was measured as described elsewhere [146,147,148].

Result interpretation and report for each method were performed as follows. For broth dilution and radial growth assays, anti-*P. insidiosum* activity of a test drug was reported as minimum inhibitory concentration (MIC), which indicates the lowest drug concentration that completely inhibits the *P. insidiosum* growth [121,130,149]. For disc diffusion assay, an inhibition zone indicated a positive drug susceptibility result [144,146]. Minimum cidal concentration (MCC) was the lowest drug concentration that showed no growth after subculturing a drug-treated organism on a drug-free SD agar [121,126,142,150]. MIC_50_ and MCC_50_ represented the drug concentration inhibiting and killing 50% of the *P. insidiosum* isolates tested. MIC_90_ and MCC_90_ depicted the same but quantified the cut-off at 90% of the test isolates [151,152,153]. The experiments were conducted in duplicate (when all 29 isolates were tested) or triplicates (when up to 10 isolates were studied). MIC and MCC were recorded after incubating the organism at 37 °C for 2 days.

### 2.4. Comparison of the In Vitro Susceptibility Assays

Disulfiram (Unidrug Innovative Pharma Technologies, India; ≥98% purity) was used as a standard substance to compare the advantages and disadvantages of broth dilution, radial growth, and disc diffusion assays for in vitro drug susceptibility testing against 3 isolates of *P. insidiosum* (i.e., Pi009, Pi050, and Pi052). Disulfiram dissolved in DMSO (Farmitalia Carlo Erba, Milano, Italy) was 2-fold diluted to a concentration range of 2–128 μg/mL for broth dilution and radial growth assays and 1000–64,000 μg/mL for disc diffusion assay. The final DMSO concentration in each disulfiram solution, including no-drug control, was 2% (*v*/*v*) in the SD medium.

### 2.5. Evaluation of Anti-P. insidiosum Activity of Potassium Iodide, Triamcinolone Acetonide, DMSO, and Ethanol

KI (Suksapan Panit, Thailand), TA (Tokyo Chemical Industry, Japan; >98.0% purity), DMSO, and EtOH (Sigma Aldrich, Germany; ≥99.9% purity) were tested against all 29 *P. insidiosum* isolates (Table 1). Drug concentration ranges were 8–128 mg/mL (in SD broth) for KI, 32–512 μg/mL (in 1% DMSO) for TA, 1–16% (in SD broth) for DMSO, and 25–99.9% (in water) for EtOH. Negative (no drug) and positive (64 μg/mL disulfiram) controls were performed in every experiment.

Broth dilution assay and hyphal plug inoculum were employed to evaluate *P. insidiosum*’s susceptibility to KI, TA, and DMSO (Table 1). The radial growth assay was also selected to test a broader range of TA (32–1024 μg/mL), KI (8–128 mg/mL), and DMSO (0.25–8%) against 10 *P. insidiosum* isolates (i.e., Pi001, Pi008, Pi009, Pi032, Pi037, Pi052, Pi054, Pi057, Pi094, and Pi105). Regarding EtOH susceptibility testing, a *P. insidiosum* hyphal plug from 10 isolates was immersed in 500 µL of 25–100% EtOH for 1, 2.5, 5, and 10 min. Each EtOH-exposed hyphal plug was washed with sterile water and subcultured on a plain SD agar plate at 37 °C for 2 days [88] before calculating a percent radial growth in reference to the EtOH-unexposed organism (control). The percent growths of all *P. insidiosum* isolates tested with different drug concentrations were compared using STATA 17 (StataCorp, TX, USA). The Kruskal Wallis test and quantile regression were performed with 95% confidence.

## 3. Results and Discussion

### 3.1. Comparison of Inoculum Types for In Vitro Drug Susceptibility

Due to its marked antimicrobial property and availability in our laboratory [111], disulfiram was an anti-*P. insidiosum* drug used for comparing the performances of three in vitro susceptibility methods (i.e., broth dilution, radial growth, and disc diffusion) and three inoculum types (i.e., hyphal plug, hyphal suspension, and zoospores) prepared from three different isolates of the organism (i.e., Pi009, Pi050, and Pi052) (Table 2). Preparation of the hyphal plug was relatively feasible, robust, and reproducible, especially when multiple isolates were simultaneously tested. On the other hand, the hyphal and zoospore suspensions were time-consuming and complicated to prepare, even from a single isolate, limiting their use in a high throughput drug susceptibility screening. The inoculum size of hyphal and zoospore suspensions can be accurately estimated using a spectrophotometer (transmittance measurement) or a light microscope (cell counting) [110,128,130,139,154]. It should be cautioned that sediment and non-homogenous suspension of the hyphae or zoospores could occur and interfere with the result reading and interpretation. Some investigators use *P. insidiosum* zoospores in drug susceptibility assays, mainly broth dilution [112,124,152]. On our hands, the preparation of zoospores usually provided a low, inadequate, and unreproducible yield (generally less than 1000 zoospores/mL). Inconsistent zoospore quantities, even prepared from the same isolate, were also observed. Due to these limitations, the current study excluded zoospore for use as an inoculum.

The hyphal plug was more versatile as it can be used in various susceptibility methods (i.e., broth dilution, radial growth, and disc diffusion). Concerning the radial growth and disc diffusion assays, it is difficult to spot and keep the hyphal suspension at the inoculated location on an agar plate because the liquid nature made it scatter or splash during the assay manipulation, transportation, and incubation. Thus, the hyphal suspension was most suitable for broth dilution performed using a multi-well plate or a set of test tubes [128,130,154]. Because zoospore is challenging to produce, some investigators replaced this inoculum type with hyphal suspension for in vitro drug susceptibility testing [130]. Preparing the hyphal suspension is more complicated than the hyphal plug but simpler than zoospore production. However, the uniform inoculum size of hyphal suspension (prepared and adjusted using a spectrophotometer) made it reliable for testing against a drug.

### 3.2. Advantages and Disadvantages of each In Vitro Susceptibility Assay

*P. insidiosum* is an oomycete whose microscopic morphology resembles filamentous fungi. A standard guideline for in vitro drug susceptibility against *P. insidiosum* has not been established. In vitro drug susceptibility analysis of *P. insidiosum* has been adapted from the standard methods of the fungi, such as CLSI M38-A2 (a procedure and interpretation guideline for broth dilution assay) [129,155,156] and CLSI M51 (a procedure and interpretation guideline for disc diffusion) [129,143]. Additionally, radial growth assay is another useful method for assessing anti-*P. insidiosum* drug activity [122,125,137,146]. Each method has advantages and limitations compared with the others (Table 2). All of these in-house assays (i.e., broth dilution, radial growth, and disc diffusion) have been used for testing the anti-*P. insidiosum* activity of a drug of interest [112,123,125,137,140,145,157]. We performed a side-by-side performance comparison of these methods and described their advantages and disadvantages below.

Broth dilution assay can be performed in test tubes or multi-well plates using any inoculum types (i.e., hyphal suspension, hyphal plug, and zoospore) and a small volume of drug solution and liquid medium (i.e., RPMI-1640 and SD broth) [111,112,130,150]. A result of the broth dilution assay can be qualitatively reported as “Growth” or “No growth” and quantitatively reported as a percent growth reduction roughly estimated by the naked eye [111,112,158]. The *P. insidiosum* growth and viability could be precisely assessed using mycelium dried weight measurement and MTT-based colorimetric analysis, respectively, as suggested by the other investigators [159,160]. Regarding radial growth assay (synonym: agar dilution assay [161,162]), preparing an agar plate for this method required a much higher drug amount (46 mg of disulfiram were needed for testing, in triplicate, against three isolates) and media volume (i.e., SD agar, vegetable extract agar, and nutrient agar) than broth dilution and disc diffusion tests (7 and 23 mg of disulfiram were respectively required) [122,125,146]. However, the radial growth assay offered a rapid and precise assessment of growth inhibition, as also described by other investigators [111,161,163]. Both broth dilution and radial growth assays can provide MICs of a drug tested against *P. insidiosum*. For disc diffusion, it has been commonly used for screening the anti-*P. insidiosum* activity of a new compound (i.e., plant extract [142,148]) and for quickly assessing the susceptibility of a clinical isolate of *P. insidiosum* against a drug of choice [45]. The significant advantage of this method over radial growth is the ability to screen and compare multiple drugs against *P. insidiosum* simply by observing a growth inhibition zone (the larger the inhibition zone, the higher the drug potency) [45,123,148]. As a downside, the disc diffusion did not provide a drug MIC unless performed an antimicrobial gradient method, such as E-test (bioMérieux, France) and MIC test strip (Liofilchem, Italy), as described by Loreto et al. [112,129,162]. Such MIC measurements are only available for certain drugs, limiting their use in the anti-*P. insidiosum* drug screening.

Different in vitro susceptibility methods or inoculum types could affect the MIC of the same drug. Some other factors, especially the medium type, could also influence the drug MIC [155,162]. Controlling such factors (i.e., using the same medium, method, and inoculum type) is essential to ensure the reliability of the in vitro drug susceptibility results. As observed in this study, broth dilution using the hyphal plug showed a higher disulfiram MIC (32–64 μg/mL) than the hyphal suspension (8 μg/mL) (Table 2). This noticeable difference could result from the hyphal plug having a piece of drug-free agar attached to one side of the *P. insidiosum* colony. This could partially prevent the organism from direct exposure to disulfiram. For the hyphal suspension, the organism was wholly immersed and exposed to the drug. Another possible explanation is that fragmented hyphae presented in the hyphal suspension (as a result of scraping the organism out of a colony) could be more vulnerable to a drug than intact organisms in the hyphal plug. When using the hyphal plug as an inoculum, broth dilution and radial growth methods demonstrated similar MICs: 32–64 μg/mL for broth dilution and 64 μg/mL for radial growth (Table 2). In the disc diffusion assay, the drug concentration in the disc is not MIC. The disc was soaked with a high disulfiram concentration (i.e., 2000 and 4000 μg/mL, depending on a *P. insidiosum* isolate tested) because the drug in the disc needed to diffuse into the plain agar and generate a drug concentration gradient. Interpretation of the disc diffusion assay relies on an organism’s inhibition zone. Suppose a markedly lower disulfiram concentration was used (i.e., 2–128 μg/mL) as in the other methods; the disc diffusion assay could fail to generate an optimal drug concentration gradient for inhibiting the *P. insidiosum* growth. We found that the inhibition zone did not appear when testing a disulfiram concentration lower than 2000 μg/mL. Nevertheless, a different MIC generated by each susceptibility method is not a concern if clinical outcome data are available for determining the method-specific drug susceptible/resistant breakpoint.

### 3.3. Evaluation of Alternative Chemicals for Anti-P. insidiosum Activity

*P. insidiosum* resists the conventional drugs designed to inhibit the fungi (i.e., ergosterol synthesis and chitin synthase inhibitors). This may be due to *P. insidiosum* possessing different cell wall components and sterol biosynthesis enzymes that are not proper targets of those antifungal drugs [1,122,164,165,166]. Searching for a new and effective anti-*P. insidiosum* agent is one of the priorities. This study evaluated the anti-*P. insidiosum* activity of several alternative chemicals (i.e., KI, TA, DMSO, and EtOH) against 29 clinical and environmental isolates of *P. insidiosum*. Because of the feasibility, robustness, and reproducibility described above, broth dilution and hyphal plug were selected as this study’s primary drug susceptibility method and inoculum type.

**Figure 1:**
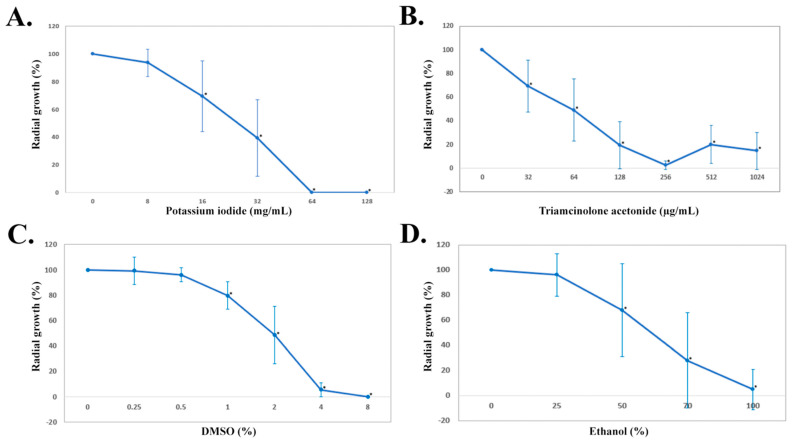
Growth reduction of *P. insidiosum* after treatment with various concentrations of potassium iodide (**A**), triamcinolone acetonide (**B**), DMSO (**C**), and ethanol (**D**). The radial growths are averaged based on 10 representative isolates of *P. insidiosum* after 2−day exposure to potassium iodide, triamcinolone acetonide, and DMSO, and 1−min exposure to absolute EtOH. An asterisk indicates a statistically significant growth reduction compared to no-drug control.

(1) Potassium iodide: MIC and MCC of KI ranged from ≤8 to 32 mg/mL and ≤8 to 64 mg/mL, respectively. MICs that inhibited at least 50% (MIC_50_) and 90% (MIC_90_) of 29 *P. insidiosum* isolates tested were 32 mg/mL. The same concentration was also defined as MCC_50_ and MCC_90_, as KI killed at least 50% and 90% of the organism population, respectively (Table 1). The susceptibility evaluation of KI against 10 *P. insidiosum* isolates using the radial growth assay showed that the organism growths were 6%, 31%, and 61% reduced after exposure to 8, 16, and 32 mg/mL of KI, respectively (Figure 1A). Moreover, the growths were inhibited entirely by 64 and 128 mg/mL of KI (Figure 1A). The statistical analysis showed that the growths were significantly reduced at concentrations of at least 16 mg/mL (*p*-value < 0.05). KI has been used to treat several fungal infections, such as sporotrichosis, basidiobolomycosis, and cryptococcosis [94,96,167]. Additionally, it has been used as a part of the pythiosis treatment [54,68,69,70,71,74,77,78,98,99]. KI, in the form of a saturated solution, is administered orally for weeks or months until the infection dissolves [15,39,67,72,74,75,97,99]. A recommended KI dose for treating an infectious disease in human adults is up to 7.5 g/day [96]. After ingestion, KI is readily absorbed, rapidly distributed in the body, and mainly excreted in the urine [94]. Long-term use of KI in human patients could lead to some adverse effects, such as iodism, potassium toxicity, and abnormal thyroid metabolism [94,96]. No hepatic and renal toxicity is noted in horses with pythiosis treated with KI for 2 months [72]. Favorable clinical outcomes following KI administration (in conjunction with other treatment modalities) have been documented in sheep, horses, and humans with pythiosis [15,39,67,72,75,78,97]. On the contrary, unresponsiveness is observed in some pythiosis cases after KI treatment [39,67,69,100].

Some investigators proposed that KI modulates the immune response (i.e., inhibiting the white blood cell chemotaxis, suppressing the oxygen intermediates production by immune cells, and exerting anti-inflammatory activity) to promote the elimination of pathogenic fungi [96,168,169,170]. The KI-dependent immune modulation is also a possible mechanism of action for eliminating *P. insidiosum*. In our in vitro study, KI appeared to directly affect *P. insidiosum* growth and viability. The chemical at 32 mg/mL (MCC_90_) killed most *P. insidiosum* isolates tested. Another piece of evidence supporting the direct effect of KI on a microorganism comes from Hiruma and Kagawa [171]. They microscopically demonstrate that KI could inhibit germination and physically destroy the fungus *Sporothrix schenckii*. However, the exact mechanism of KI’s antifungal action still needs further investigation. Apart from KI, sodium iodide is another iodine salt infrequently used in treating pythiosis, providing uncertain clinical outcomes [84,172,173].

(2) Triamcinolone acetonide: TA is a synthetic glucocorticoid widely used for treating autoimmune diseases and cancers [102,103,174,175]. TA is an immunomodulator that could activate macrophages, increase an interleukin-10 level, and reduce eosinophils and immunoglobulin E antibodies [176,177]. Intramuscular injection of TA, as a monotherapy, cured several horses with cutaneous pythiosis [68,79,80,81,82]. This study evaluated TA for its antimicrobial effect against *P. insidiosum*. The broth dilution method demonstrated that the range of TA MICs and MCCs spanned from ≤32 to >512 µg/mL. MIC_50_, MIC_90_, MCC_50_, and MCC_90_ of TA were greater than 512 µg/mL (the maximal TA concentration used in broth dilution; Table 1). Only one isolate (Pi050) from a cutaneous pythiosis patient in the United States exhibited MCC less than 32 µg/mL. Five of 6 TA-sensitive isolates (i.e., Pi060, Pi057, Pi075, Pi077, and Pi094) showed MCCs of 128–256 µg/mL, but their growths resumed when incubating in an agar plate with a higher TA concentration (i.e., 512 µg/mL). Colonies of most TA-insensitive organisms (MIC and MCC > 512 µg/mL) that exposed to TA at 512 µg/mL were larger than at 256 µg/mL. To confirm this observation, the radial growth assay was used to evaluate TA (concentration range: 32–1024 µg/mL) for its antimicrobial activity against 10 representative isolates of *P. insidiosum*. Similar results were observed, in which the growths were significantly reduced in a dose-dependent manner until the organisms resumed growing at a TA concentration higher than 256 µg/mL (Figure 1B). This paradoxical phenomenon has been described as the Eagle effect, in which an organism regrows at the drug concentration above MCC [178,179]. Although the underlying mechanism of the Eagle effect is unclear, it could result from drug impurity, reduction of autolytic activity, increase in drug-inactivated enzyme, and reduction of reactive oxygen species [178,180]. In our case, drug purity should not be the cause since a high-purity TA (98%) was used. However, we observed limited solubility of TA in a solvent at a concentration greater than 256 µg/mL, which led to drug precipitation and, thus, lower-than-expected antimicrobial drug activity. Although the mechanism of anti-*P. insidiosum* effect of TA is unknown, we proposed that the direct antimicrobial effect (especially for TA-sensitive isolates) and the immunomodulatory properties of TA could contribute to the elimination of *P. insidiosum*.

(3) Dimethyl sulfoxide: DMSO has been used as a solvent, antioxidant, anti-inflammatory, and antimicrobial agent [105,106,109,181]. Regarding its antimicrobial activity, DMSO can inhibit some bacteria (i.e., *Escherichia coli* and *Pseudomonas aeruginosa*) and fungi (i.e., *Botrytis cinerea*, dermatophytes and *Candida albicans*) [181,182,183,184]. DMSO was intravenously administered, in conjunction with amphotericin B, in 15 horses with cutaneous pythiosis [87]. It was also topically applied to the post-surgical skin lesion of 17 affected horses [86]. DMSO can promote recovery in such infected horses [86,87]. In this study, we elaborated on the clinical finding by investigating the in vitro antimicrobial activity of DMSO against 29 *P. insidiosum* isolates. Based on the broth dilution method, DMSO MICs and MCCs ranged from 2 to 8% (*v*/*v*). MIC_50_, MIC_90_, MCC_50_, and MCC_90_ were all at 8% DMSO (Table 1). The radial growth assay was also used to test 10 representative *P. insidiosum* isolates against various DMSO concentrations (i.e., 0.25%, 0.5%, 1%, 2%, 4%, and 8%) and demonstrated dose-dependent growth reductions (i.e., 0.8%, 4.0%, 20.3%, 51.4%, 94.6% and 100.0%, respectively; Figure 1C). Compared with the no-drug control, the organism growths were significantly reduced following the exposure to at least 1% of DMSO (*p*-value < 0.05; Figure 1C). As shown here, DMSO concentrations, particularly down to 2%, can kill *P. insidiosum* (Table 1). The anti-*P. insidiosum* mechanism of DMSO action might be the same as described in other pathogens, such as increasing the membrane permeability, altering the expression of cell wall protein, and changing the enzymatic activity [108,185]. Various DMSO concentrations have been applied for many medical and scientific purposes. For example, 90% DMSO is commonly used in skin diseases [186], and 50% DMSO shows a treatment benefit in eye diseases and interstitial cystitis [187,188]. For a laboratory experiment involving cell culture, 10% DMSO is used as a cryo-preservative agent [189]. Depending on an administered dose and route, several adverse effects of DMSO could be noticed, for example, retinal apoptosis, hemolysis, fibrinogen precipitation, cardiac arrhythmia, and genetic changes [189,190,191].

(4) Ethanol: EtOH is an antiseptic agent [116]. Previous reports show that, when used locally as an adjunctive treatment with surgery and other medications, absolute EtOH can lead to favorable clinical outcomes in treating a small group of ocular pythiosis patients [88,89,90]. In the current study, various EtOH concentrations (i.e., 25%, 50%, 70%, and 100%) were tested for their antimicrobial effect against 10 *P. insidiosum* isolates at several time points (i.e., 1, 2.5, 5, and 10 min). Compared with the no-drug control, the *P. insidiosum* growths were significantly reduced after 1 min exposure to 50% (*p*-value = 0.03), 70% (*p*-value < 0.001), and 100% (*p*-value < 0.001), but not 25% (*p*-value = 0.53) EtOH (Figure 1D). Like the other EtOH concentrations, 25% EtOH inhibited the organism’s growth more significantly when the exposure time was longer, such as 5 min (*p*-value = 0.01) and 10 min (*p*-value < 0.001) (Supplementary Figure S1). Taken together, EtOH can inhibit the organism in a dose- and time-dependent manner: higher concentration and longer exposure time enhance growth suppression. In a clinical setting, Agarwal et al. have topically applied absolute (100%) EtOH at the infection site of a few ocular pythiosis cases for 1 min, resulting in favorable treatment response [88]. We augmented their finding by challenging 29 isolates of *P. insidiosum* with absolute EtOH for 1 min. The result showed complete growth inhibition in 83% of all isolates tested (n = 24; Table 1). Regarding the mechanism of action, EtOH affects, for example, fungal organisms in various ways that lead to abnormal mitotic spindle, abnormal morphology, and reduced cell membrane permeability [192]. EtOH toxicities (i.e., cell lysis, inducing apoptosis, and suppressing cell proliferation) are a concern when using this chemical [193,194]. Nevertheless, EtOH could be a potential alternative agent for managing a local *P. insidiosum* infection.

## 4. Conclusions and Perspectives

Novel, alternative, or repurposed drugs effective against *P. insidiosum* (an orphan but highly virulent pathogen) are urgently needed. Assessing the antimicrobial activity of a drug of interest requires a standardized in vitro susceptibility test. However, no such test is available for this organism. Several in-house drug susceptibility tests (i.e., broth dilution, disc diffusion, and radial growth assays) have been established, some of which adapted the standard methods (i.e., CLSI M38-A2 and CLSI M51 protocols) designed for fungi. Hyphal plug, hyphal suspension, and zoospores are inoculum types commonly used in the drug susceptibility assessment for *P. insidiosum*. We demonstrated that each method has advantages and limitations compared to the others (Table 2). Selecting an assay and inoculum type depends on material availability; the experience of a laboratory worker; and the number of isolates and drugs to be tested

In this study, we employed the hyphal plug (served as an inoculum) and a combination of broth dilution and radial growth methods to screen and validate the anti-*P. insidiosum* activities of four chemicals (i.e., KI, TA, DMSO, and EtOH). Other investigators have preliminarily reported these chemicals as effective agents in treating pythiosis. We augmented their findings by extensively testing these chemicals against 29 genetically diverse isolates of *P. insidiosum* (Table 1). The results show that KI, TA, DMSO, and EtOH possessed an antimicrobial effect against *P. insidiosum* in a dose- or time-dependent manner. This information suggests that these chemicals could be potentially applied systematically (i.e., KI and TA) or locally (i.e., DMSO and EtOH) to treat pythiosis. The mechanism of action of these chemicals needs to be elucidated to understand how they work.

There is no standardized method for in vitro drug susceptibility analysis of *P. insidiosum*. Future attempts should emphasize standardizing the drug susceptibility methods, including determination of susceptibility and resistant breakpoints, for *P. insidiosum*, so healthcare workers can confidently read and interpret a result for selecting the most effective drug against the pathogen.

## Figures and Tables

**Table 1:** Drug susceptibility analyses (i.e., determining minimal inhibitory (MIC) and cidal (MCC) concentrations and percent growth reduction) of potassium iodide (KI), triamcinolone acetonide (TA), dimethyl sulfoxide (DMSO), and ethanol (EtOH) tested against 29 *P. insidiosum* isolates. The 10 *P. insidiosum* isolates (indicated by an asterisk) were selected to test each chemical with radial growth assay. Broth dilution assay and hyphal plug inoculum are used to evaluate *P. insidiosum*’s drug susceptibility. MIC_50_ and MCC_50_ represent drug concentrations inhibiting and killing 50% of the isolates tested. MIC_90_ and MCC_90_ are the drug concentrations that inhibit and kill 90% of the studied population.

Strain ID	Laboratory ID	Host (Tissue)	Country of Origin	Clade	KI (mg/mL)		TA (µg/mL)		DMSO (%)		EtOH
					MIC	MCC	MIC	MCC	MIC	MCC	Growth Reduction (%)
Pi001 *	CBS 578.85	Equine	Costa Rica	I	32	64	128	>512	8	8	100
Pi002	CBS 579.85	Equine	Costa Rica	I	32	32	128	>512	4	4	100
Pi008 *	CBS 580.85	Equine	Costa Rica	I	32	32	>512	>512	8	8	100
Pi009 *	CBS 101555	Equine	Brazil	I	32	32	>512	>512	8	8	100
Pi010	ATCC 200269	Human (skin)	USA	I	16	32	128	>512	8	8	94
Pi060	EQ04	Equine	Brazil	I	32	32	128	256	8	8	100
Pi074	P45	Dog	Thailand	I	16	32	>512	>512	8	8	100
Pi012	SIMI 149-41	Human (artery)	Thailand	II	16	16	>512	>512	8	8	100
Pi020	MCC 18	Human (eye)	Thailand	II	16	16	>512	>512	8	8	100
Pi023	MCC 10	Human (gut)	Thailand	II	32	32	>512	>512	4	8	100
Pi025	P19	Human (eye)	Thailand	II	16	32	>512	>512	4	8	100
Pi032 *	P34	Human (eye)	Thailand	II	32	32	>512	>512	8	8	100
Pi033	P36	Human (artery)	Thailand	II	32	32	>512	>512	8	8	100
Pi035	Pi-S	Human (artery)	Thailand	II	32	32	>512	>512	8	8	100
Pi036	ATCC 64221	Equine	Australia	II	32	32	>512	>512	8	8	100
Pi037 *	ATCC 28251	Equine	New Guinea	II	32	32	>512	>512	8	8	100
Pi038	CBS 101039	Human (eye)	India	II	32	32	>512	>512	4	8	100
Pi042	CR02	Environment	Thailand	II	32	32	>512	>512	8	8	100
Pi052 *	P38	Human (artery)	Thailand	II	32	32	>512	>512	8	8	88
Pi053	P39	Equine (nose)	Thailand	II	32	64	>512	>512	8	8	100
Pi054 *	P40	Human (artery)	Thailand	II	32	32	>512	>512	8	8	100
Pi055	P41	Human	Thailand	II	16	16	>512	>512	4	4	100
Pi050	ATCC 90586	Human (skin)	USA	III	≤8	≤8	≤32	≤32	2	2	100
Pi057 *	P43	Human	Thailand	III	16	32	256	256	8	8	69
Pi075	P46	Human (eye)	Thailand	III	16	32	256	256	4	8	100
Pi077	P48	Environment	Thailand	III	16	16	128	128	4	8	91
Pi089	KCB 09	Environment	Thailand	III	16	32	>512	>512	8	8	22
Pi094 *	P52	Human (eye)	Thailand	III	16	32	128	256	4	8	100
Pi105 *	60P 21-1	Human	Thailand	III	32	32	128	>512	4	4	100
Range					≤8–32	≤8–64	≤32–>512	≤32–>512	2–8	2–8	22–100
MIC_50_ and MCC_50_					32	32	>512	>512	8	8	Not applicable
MIC_90_ and MCC_90_					32	32	>512	>512	8	8	Not applicable

**Table 2:** Side-by-side performance comparison of 3 in vitro drug susceptibility methods and 3 inoculum types for assessing anti-*P. insidiosum* activity of disulfiram. Abbreviations: MIC, minimal inhibitory concentration; ND, not conducted due to the zoospore production providing inadequate yield for the in vitro susceptibility assays.

In Vitro Susceptibility Methods	Inoculum Types	MIC (μg/mL) or Inhibition Zone (mm) of Disulfiram against 3 *P. insidiosum* Isolates			Advantages/Benefits	Disadvantages/Limitations
		Pi009	Pi052	Pi050		
Broth dilution	Hyphal plug	64	32	32	The method is feasible for routine use; small drug amount is required.	Only “growth” or “no growth” is reported; growth cannot be directly quantified.
	Hyphal suspension	8	8	8	Inoculum size is quantifiable; small drug amount is required.	Inoculum preparation for multiple isolates is unfeasible and complicated; only “growth” or “no growth” is reported; growth cannot be directly quantified.
	Zoospore	ND	ND	ND	Inoculum size is quantifiable.	Inoculum preparation for multiple isolates is time-consuming, unfeasible, and complicated; production yield (zoospore numbers) is low and inadequate for testing; only “growth” or “no growth” is reported; growth cannot be directly quantified.
Radial growth	Hyphal plug	64	64	64	The method is feasible for routine use; growth can be directly quantified.	Large drug amount is required; the method is time-consuming for testing many isolates.
Disc diffusion	Hyphal plug	1.83 ^a^ (4000 ^b^)	2.00 ^a^ (2000 ^b^)	2.67 ^a^ (4000 ^b^)	The method is suitable for simultaneously testing multiple drugs; small drug amount is required.	MIC cannot be directly measured.

Footnotes: ^a^ The size of inhibition zone in mm. ^b^ Concentration of disulfiram loaded on the disc.

## Data Availability

Not applicable.

## Supplementary Materials

The following supporting information can be downloaded at: https://www.mdpi.com/article/10.3390/jof8111116/s1, Figure S1: Growth reduction of *P. insidiosum* following the treatment with various ethanol concentrations at several time points.
