# Supplementary material for: Selection of an Appropriate In Vitro Susceptibility Test for Assessing Anti-Pythium insidiosum Activity of Potassium Iodide, Triamcinolone Acetonide, Dimethyl Sulfoxide, and Ethanol
Source: J Fungi (Basel). 2022 Oct 24;8(11):1116. doi: 10.3390/jof8111116 (PMC9692648; doi:10.3390/jof8111116)
Supplement: Supplementary file 1 [file jof-08-01116-s001.zip › jof-1930550-supplementary.pdf]

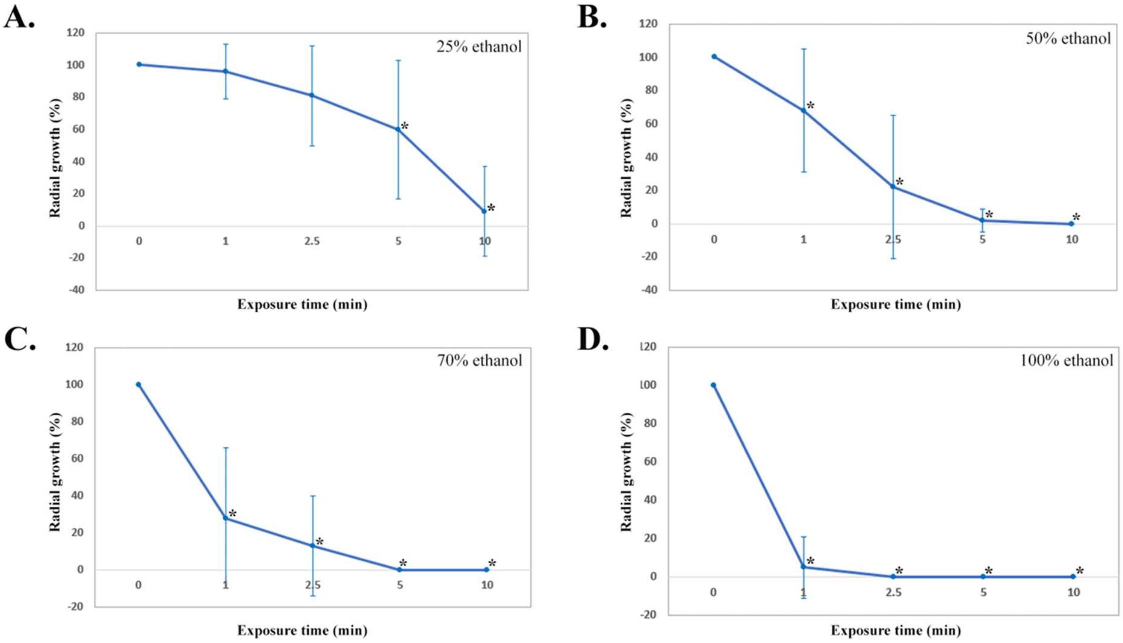

Supplementary Figure S1. Growth reduction of *P. insidiosum* following the treatment with various ethanol concentrations at several time points. The radial growths are calculated based on 10 representative *P. insidiosum* isolates after exposure to 25% (**A**), 50% (**B**), 70% (**C**), and 100% (**D**) ethanol for 1, 2.5, 5, and 10 min. An asterisk indicates a statistically significant growth reduction compared to no-drug control.
